# Function of Tumor Suppressors in Resistance to Antiandrogen Therapy and Luminal Epithelial Plasticity of Aggressive Variant Neuroendocrine Prostate Cancers

**DOI:** 10.3389/fonc.2018.00069

**Published:** 2018-03-15

**Authors:** Rama Soundararajan, Ana M. Aparicio, Christopher J. Logothetis, Sendurai A. Mani, Sankar N. Maity

**Affiliations:** ^1^Department of Translational Molecular Pathology, The University of Texas MD Anderson Cancer Center, Houston, TX, United States; ^2^Department of Genitourinary Medical Oncology, The University of Texas MD Anderson Cancer Center, Houston, TX, United States

**Keywords:** tumor suppressors, neuroendocrine prostate cancer, lineage plasticity, therapy resistance, epithelial-to-mesenchymal transition

## Abstract

Combined loss of tumor suppressors (TSPs), PTEN, TP53, and RB1, is highly associated with small cell carcinoma of prostate phenotype. Recent genomic studies of human tumors as well as analyses in mouse genetic models have revealed a unique role for these TSPs in dictating epithelial lineage plasticity—a phenomenon that plays a critical role in the development of aggressive variant prostate cancer (PCa) and associated androgen therapy resistance. Here, we summarize recently published key observations on this topic and hypothesize a possible mechanism by which concurrent loss of TSPs could potentially regulate the PCa disease phenotype.

Small-cell prostate carcinoma (SCPC) is a common lethal variant of prostate cancer (PCa) that is androgen receptor (AR) negative and thus represents a mechanism for escape from the potent antiandrogen treatments. The presence of SCPC morphologic characteristics predicts for a distinct clinical course with a dismal prognosis, despite a heightened sensitivity to chemotherapy (1–4). Although often unrecognized, SCPC is frequently found on repeat biopsies of previously diagnosed adenocarcinoma of the prostate during castration-resistant PCa (CRPC) (5, 6) and is present in 10–20% of autopsies of patients who die of the disease (7–10). SCPC also phenotypically overlaps with neuroendocrine prostate cancer (NEPC), and clinicopathologic characteristics of SCPC/NEPC are generally associated with worse progression-free survival (3, 11–14).

Molecular characterization of human CRPC and SCPC/NEPC tumors showed that concurrent alteration of three tumor suppressors (TSPs), PTEN, TP53, and RB1, in which loss of copy numbers of both PTEN and RB1, and loss of copy number as well as missense mutations of TP53 genes are highly enriched in SCPC/NEPC tumors (Table 1) (15–24). Conditional deletion of the TSP genes in mouse luminal epithelial cells of prostate demonstrated new insights into the functional role of these TSPs in therapy resistance (25–28). Studies in mouse models as well as in human PCa cells showed that deletion of both RB1 and TP53 resulted in development of aggressive prostate tumors that are resistant to anti-androgen treatment (27, 28). Deletion of all three TSPs—RB1, TP53, and PTEN—resulted in development of lethal PCa and significantly increased cancer-related death and metastasis in mice (27). Expression analysis of mouse tumors showed that loss of TSPs, induced epithelial lineage plasticity with induction of neuroendocrine markers but loss of AR and epithelial makers. The study further showed that loss of RB1 plays a deterministic role in inducing lineage plasticity when combined with the loss of PTEN and TP53. In contrast, deletion of RB1 by itself showed no impact in prostate tumor development (Table 1).

**Table 1:** Impact of combined loss of three tumor suppressors—PTEN, TP53, and RB1—in human and mouse models of prostate cancer.

Tumor suppressors status	Human prostate cancer: patient tumors and patient tumor-derived xenografts (PDXs)		Mouse model prostate cancer; conditional deletion of tumor suppressor genes in mouse prostate epithelium^c^	Reference
	CRPC: adenocarcinoma; epithelial markers positive; AR positive^a^	SCPC/NEPC: morphological heterogeneous; neuroendocrine and proneural markers positive; AR negative^b^		
RB1 loss	10–30%	70–90%	No prostate cancer	(18–24, 27, 28)
Combines lossRB1 + TP53	5–10%	30–40%	NEPC-like tumor and castration resistance; decreased expression of AR	
Combined lossPTEN + RB1 + TP53	4–6%	30–35%	Aggressive prostate cancer; castration resistance; short survival; loss of AR expression	

*^a^TSPs status of CRPC is reported from Grasso et al. (18), Robinson et al. (21), and Abida et al. (24)*.

*^b^TSPs status of SCPC/NEPC is reported from Tzelepi et al. (19), Tan et al. (20), Aparicio et al. (22), and Beltran et al. (23)*.

*^c^Impact of TSPs in mouse models are reported from Ku et al. (27) and Mu et al. (28)*.

The SCPC phenotype can be morphologically heterogeneous and is often described as mixed tumors harboring both typical adenocarcinoma and NEPC, suggesting that the SCPC/NEPC tumors constitute multiple cell types or cells in multiple distinct stages of differentiation. Studies in patient tumor-derived xenografts as well as in human tumors showed that the SCPC phenotype is associated with induction of the pro-neural developmental program, including expression of master transcription factors such as ASCL1, NEUROD1, and BRN2 that determine proneural differentiation (22, 29, 30). Several investigations reported expression of stem and basal cell markers in SCPC/NEPC tumors, suggesting that these tumors might originate from basal cells of the prostate (31, 32). However, analysis of the mouse tumors with conditional deletion of TSPs in luminal epithelial cells strongly indicated that combined loss of TSPs induces lineage plasticity resulting in loss of AR expression but gain of stemness master transcription factor such as SOX2, along with upregulation of NEPC markers (27, 28). Importantly, lineage-tracing experiments demonstrated that anti-androgen therapy, by itself, could induce trans-differentiation of luminal epithelial cells into prostate NEPC-like cells lacking functional PTEN and TP53 TSPs (33). Of note, previous studies of compound deletions of PTEN and TP53 TSPs in mice, wherein gene inactivation was done by a constitutive prostate-specific Cre driver, showed that combined TSP deletions led to development of invasive CRPC phenotype (34, 35). A more recent publication, however, used regulated inducible Cre driver in adult mice that specifically deleted PTEN and TP53 genes in prostate luminal epithelial cells, which allowed for a more detailed study about the role of PTEN and TP53 TSPs in therapy-induced NEPC transdifferentiation (33). Taken together, these studies suggest that cellular reprogramming or trans-differentiation could be a central mechanism potentiating resistance to anti-androgen therapy, in which loss of functional TSPs facilitates alterations in the epithelial lineage. This plasticity is observed in the switch to pro-neural and pluripotent-like cancer cells that are no longer dependent on AR activity.

Role of TSPs in cellular reprogramming or lineage plasticity is a nascent concept. Although the individual function of each TSP has been studied extensively for many years, the impact of combined loss of TSPs in specific cellular pathways that can be linked to therapy-resistance remains largely unclear. Importantly, TP53 loss or RB1 loss events in prostate tumors are not currently druggable. The proteins p53 and Rb1 encoded by TP53 and RB1 genes, respectively, are known to function as nuclear transcriptional activator and repressor, respectively. Loss of p53 function inhibits expression of downstream genes that are direct targets of p53 protein (36), whereas loss of Rb1 results in activation of E2F transcription factors regulating cell cycle genes, the pluripotency (transcription) factor SOX2, as well as heterochromatin regulators (27, 37–39, 40, 41). The SCPC/NEPC tumors also express mutant p53 proteins, which although cannot bind to promoters of p53 target genes, can however indeed regulate transcription *via* activation of distinct transcription factors or chromatin regulators driving oncogenic pathways (42, 43).

Induction of lineage plasticity due to loss of TSPs suggests that these TSPs likely play a role in maintaining the fundamental state of epithelial differentiation in the prostate. We propose that loss of TSPs in luminal epithelial cells induces epigenetic and transcriptional reprogramming resulting in differential expression of master transcription factors allowing lineage plasticity and thereby, the opportunity for trans-differentiation (Figure 1). Once the stage is set for this cellular fluidity, further molecular “hits” such as epithelial-to-mesenchymal transition (EMT), which can be induced by anti-androgen therapy (44), could then propel transition to SCPC/NEPC. The term EMT is used to describe profound cell biological transitions that convert “epithelial” tissue-resident cells into morphologically and functionally distinct “mesenchymal or mesenchymal-like” cells harboring increased migratory and invasive properties facilitating disease recurrence and progression. The genes expression analyses of both human and mouse prostate tumors demonstrate increased expression of master EMT transcription factors (FOXC2, ZEB1, SNAIL) (45, 46). These factors are known to play a critical role in the inhibition of the epithelial-specific transcriptional program, including inhibiting expression of AR, and instead inducing expression of mesenchymal markers.

**Figure 1:**
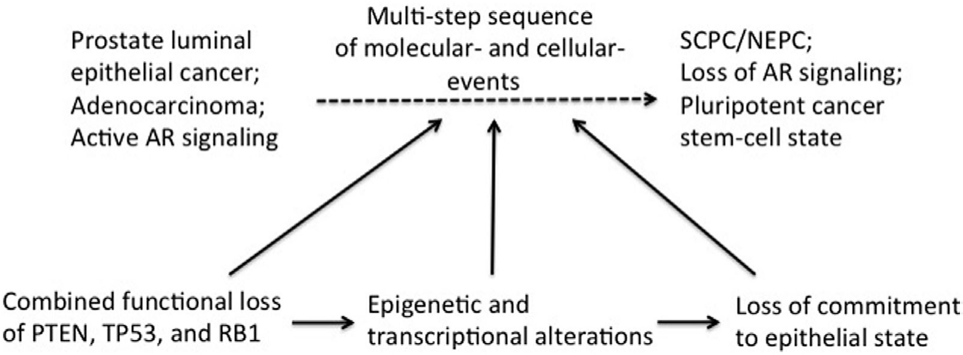
Induction of transdifferentiation of epithelial prostate cancer cells may be brought about in multiple steps involving sequential loss of tumor suppressors (TSPs) function and pluripotency/plasticity events. Combined functional loss of TSPs (PTEN, RB, and P53), epigenetic and transcriptional modifiers, as well as pluripotency and stemness events have each been linked to the altered cellular differentiation process during prostate tumor progression. It is conceivable that these events have a preferential order of occurrence in the history of the tumor development, with each event contributing partially to tumor progression and also setting the stage for the next subsequent event. Combined TSP loss is perhaps an early event in this context, facilitating ensuing complex changes in the epigenome/transcriptome of the early “primed” tumor cell. Together with powerful cell fate modifiers [such as epithelial-to-mesenchymal transition (EMT) and EMT-induced stemness], the changing tumor cell would then be equipped with pluripotency traits needed to fuel self-sustenance.

Interestingly, loss of TP53 and RB1 TSPs is also highly associated with small-cell lung cancer (SCLC), a histological subtype representing nearly 15% of all lung cancers (47). SCLC expresses various neuroendocrine markers including the proneural master transcription factors, ASCL1 and NEUROD1 (48). The non-small-cell lung cancer (NSCLC) subtype, accounting for the majority of the cases, includes adenocarcinoma and is often treated with tyrosine kinase inhibitors targeting epidermal growth factor receptor (EGFR)-activating mutations. SCLC can develop as part of a resistance mechanism to targeted EGFR therapy. Analysis of SCLC tumors utilizing patient tumors and mouse models suggest that the SCLC phenotype can be developed due to transformation or trans-differentiation of NSCLC adenocarcinoma, as a result of RB1 inactivation and/or loss of EGFR expression, as recently reviewed extensively (49, 50). In this regard, it is conceivable that development of both SCPC/NEPC and SCLC can be driven by similar cellular mechanisms involving cell fate changes (47, 50).

Resistance to antiandrogen therapy can also emerge as an AR-independent mechanism without development of the SCPC/NEPC phenotype, as recently revealed in two publications (51, 52). In this case, activation of the fibroblast growth factor and mitogen-activated protein kinase pathways can drive CRPC tumor growth in the absence of both AR and SCPC/NEPC markers (termed as “double negative”) (51). Another mechanism includes activation of the gastrointestinal (GI) lineage transcriptional program in CRPC, in which the resistant tumor cells utilize two hepatocyte nuclear factors (HNF1A and HNF4G), that drive an alternate lineage-specific program due to suppression of AR signaling (52). Since AR plays a key role in driving/sustaining the prostate luminal epithelial lineage program, loss of AR or activation of GI lineage transcription events would alter the luminal epithelial cell fate, in turn resulting in the onset of the AR-independent CRPC phenotype. The specific epigenetic mechanism/s and/or genomic deletion that might be initiating the AR-independent tumors without SCPC/NEPC phenotype are yet to be identified.

In summary, concurrent loss of the TSPs—PTEN, RB1, and TP53—permits powerful cell-fate adaptations (*via* altered epigenetic and transcriptional rewiring) that together allow the tumor cell a new capacity to transition to a distinct cell fate. Following this necessary and initial event, cell biological processes like EMT determine the ultimate phenotype of these altered tumor cells along with rapid development of therapy resistance, much like in other solid tumors such as breast or lung cancers. We speculate that continued expression of EMT and/or stem-cell factors would sustain the SCPC/NEPC tumors in a pluripotent yet reversible state. The challenge lies in identifying unique and significant modifiers of epithelial lineage plasticity. Understanding the contribution of these “second” hit events such as epigenetic and transcriptional alterations may offer workable therapeutic opportunities overcoming resistance to antiandrogen therapy.

## Author Contributions

SM and RS developed conceptual framework and wrote the manuscript. AA, CL, and SAM developed conceptual framework.

## Conflict of Interest Statement

The authors declare that the research was conducted in the absence of any commercial or financial relationships that could be construed as a potential conflict of interest.
